# A method to enhance spatial resolution of a 2D ion chamber array for quality control of MLC

**DOI:** 10.1120/jacmp.v12i4.3456

**Published:** 2011-11-15

**Authors:** Rogelio Díaz Moreno, Daniel Venencia, Edgardo Garrigo, Yakov Pipman

**Affiliations:** ^1^ Departamento de NeurocirugÍa Instituto de NeurologÍa y NeurocirugÍa La Habana Cuba; ^2^ Instituto Privado de Radioterapia Córdoba Argentina; ^3^ Department of Radiation Medicine NSLIJ Health System New Hyde Park New York

**Keywords:** multileaf collimator, quality control, 2D detector array, partial volume response of detector

## Abstract

This work introduces a new method for verifying MLC leaf positions with enough spatial resolution to replace film‐based methods in performing QA tests. It is implemented on a 2D ion chamber array, and it is based on the principle of varying signal response of a volumetric detector to partial irradiation. A PTW 2D‐ARRAY seven29 (PTW‐729 2D) array was used to assess a Siemens OPTIFOCUS MLC. Partial volume response curves for chambers in the array were obtained by irradiating them with the leaves of the MLC, progressively covering varying portions of the chambers correlated with the leaf positions. The readings from the array's chambers are processed with an in‐house program; it generates a reference response that translates readings into leaf positions. This principle allows discriminating errors in pairs of opposing leaves that could combine to cancel their detection with other tools.

Patterns of leaf positions, similar to the Bayouth test but with different, purposefully introduced errors, were generated and used to test the effectiveness of the method. The same patterns were exposed on radiographic film and analyzed with the RIT software for validation. For four test patterns with a total of 100 errors of ±1 mm, ±2 mm and ±3 mm, all were correctly determined with the proposed method. The analysis of the same pattern with film using the Bayouth routine in the RIT software resulted in either somewhat low true positives combined with a large fraction of false positives, or a low true positive rate with a low false positive ratio, the results being significantly affected by the threshold selected for the analysis.

This method provides an effective, easy to use tool for quantitative MLC QA assessment, with excellent spatial resolution. It can be easily applied to other 2D arrays, as long as they exhibit a partial volume detector response.

PACS number: 87.55.Qr

## I. INTRODUCTION

The use of multileaf collimators (MLC) in radiotherapy calls for quality control procedures that guarantee their correct functioning.^(^ ^1^ ^)^ Accurate positioning of leaves constitutes a critical parameter in treatment delivery, especially in intensity modulated radiation therapy (IMRT).^(^ ^2^ ^,^ ^3^ ^)^ The literature describes different procedures to evaluate the mechanical accuracy and reproducibility of MLCs, which are mainly based on irradiation of radiographic films with fields of various geometries.^(^ ^4^ ^)^ Recently, several authors have pointed out the current trend to move to digital imaging. This trend has resulted in a decline in the availability of films and chemical processors,^(^ ^5^ ^,^ ^6^ ^)^ which led us to look for alternative methods for this type of control.

2D arrays of detectors could be considered a solution for replacing radiographic films;^(^ ^7^ ^)^ however, their low spatial resolution limits their applicability in resolving position errors in the order of few millimeters. The following work shows that this limitation can be solved by taking into account the fact that partial coverage of a volumetric detector by a radiation beam gives a response proportional to the irradiated volume, as described by Yang et al.^(^ ^8^ ^)^ Spezi et al.^(^ ^9^ ^)^ studied the response of a 2D ion chamber array when radiation fields gradually increase the coverage of the detector area, and found a close correspondence between detector coverage and its response.

The MLC_Fastchecker software^(^ ^10^ ^)^ is a tool developed for the 2D ion chamber array PTW‐729 to detect leaf positioning errors, associated with failures in the positioning of adjacent fields. However, this method does not allow determining which leaf of an opposing pair causes the error, nor does it guarantee that two errors of opposite effects will not go undetected.

The aim of this work is to implement a MLC quality control method that allows quantifying the error in individual leaf positioning using a 2D ion chamber array based on the concept of ion chamber partial volume response.

## II. MATERIALS AND METHODS

A PTW‐729 2D ion chamber array (PTW‐Freiburg, Germany), which has 729 detectors on a uniform matrix of 27 rows and 27 columns, was used. Each ionization chamber has an active cubic volume of 5 mm side. The chambers are separated by 5 mm septa, establishing a maximum detection area of $27\times 27\;{\text{cm}}^{2}$. Detectors are covered with a 5 mm depth PMMA layer. Several authors have studied the sensitivity and reproducibility parameters of this array and the results obtained are comparable with similar, commonly used detectors.^(^ ^7^ ^,^ ^9^ ^)^ The VeriSoft software (PTW‐Freiburg, Germany) was used to record radiation dose readings. The array was optically aligned with the MLC so that each leaf corresponded to one row of detectors.

A 6 MV beam from a Primus linear accelerator (Siemens Medical Solution, Inc., Concord., CA) equipped with a 82 leaf MLC OPTIFOCUS (Siemens Medical Solution, Inc., Concord, CA) was used. The MLC leaves have a 1 cm wide projection at the isocenter, a 10 cm overtravel over the collimator axis, and a stated inaccuracy of leaf positioning not greater than 1 mm.^(^ ^11^ ^)^

**Figure 1:**
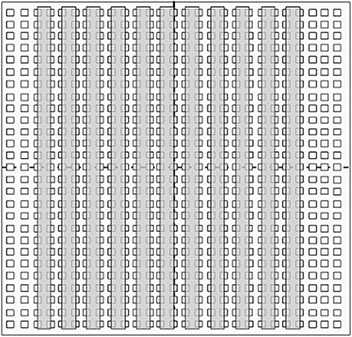
Pattern of rectangular radiation beams on the 2D detector array.

**Figure 2:**
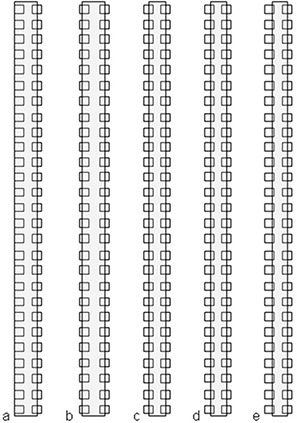
Variations of the sequential positions of the left side of the rectangular field used to calibrate the detector response of the left column of chambers.

First, a regular test pattern of 11 rectangular fields of 1cm×27cm with intervening unirradiated bands of 1 cm was generated; the area thus covered is 21×27 cm, similar to the pattern studied by Bayouth et al.,^(^ ^4^ ^)^ as shown in Fig. 1. In order to obtain the data for the partial volume response curve for each ion chamber of the 2D array, eight additional calibration patterns were then created based upon variations of the first pattern. One side of each rectangular field was shifted in steps of 1 mm, over a range of plus and minus 2 mm, so that the fraction of each detector covered by the radiation beam under its variable side would vary, as shown in Fig. 2(a)–2(e). The other side of the rectangular field was kept in its fixed position over the center of the complementary detectors column; then, the fixed and variable sides were reversed.

The response of each detector to different positions of the leaves for a constant number of monitor units in each field was recorded in the dose file returned by the PTW‐729. A calibration file was created from the response of the detector readings under the variable field edge, normalized by the average reading of all the detectors in the adjacent column which is kept under the fixed field edge. As the fixed and variable field edges were reversed, the detectors in both sides were thus calibrated.

**Figure 3:**
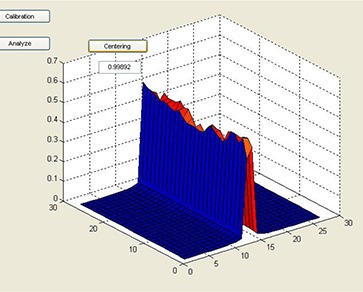
View of AURIL's tool and quantification index for device centering.

A software program (AURIL, AUtomatic Radiotherapy program for Information of Leaves) was developed in MATLAB environment (The MathWorks, Natick, MA). The software reads the files containing the dose readings resulting from the irradiation of the calibration patterns. This information is arranged in a suitable matrix that contains the normalized response of the detectors to varying degrees of partial volume irradiation, as described above. For subsequent MLC regular test patterns, AURIL allows evaluating deviations corresponding to individual leaf positioning errors, assuming a linear dependence between position and detector response. Since a strict alignment of the PTW‐729 device with the collimator/leaf axis is critical, a tool was developed in AURIL that calculates the mean readings ratio for the detectors under the left and right sides of the central rectangular field of the regular band pattern (Fig. 3). A ratio between 0.97 and 1.03 was considered satisfactory.

For all the subsequent tests, the PTW‐729 device was irradiated at 100 cm SAD and 15 mm of water equivalent material (RW3, PTW‐Freiburg, Germany) as buildup and 90 MU for each field of the patterns described above. Because of the PTW‐729 size, only the 27 central leaves of the MLC were studied; the width limitation was given by the maximum overtravel of the MLC. For validation of the sensitivity of this method and the associated AURIL software program, four verification test patterns were created and irradiated on the PTW‐729 after the calibration process. Each of these verification test patterns included predetermined, arbitrarily chosen leaf position errors of 1 mm, 2 mm and 3 mm, as shown in Table 1.

**Table 1:** Summary of results of errors introduced into the validation plans and detected by AURIL software.

Value of the introduced error (mm)	±1	±2	±3
Number of introduced errors	84	32	2
Numbers of errors correctly detected	84	32	2
Mean absolute values of the detected errors (mm)	0.97	1.84	–
Standard deviation (mm)	0.17	0.16	–
Binned values (mm)	0.6–1.4	1.5–2.3	2.7–2.8

All these tests were carried out also with X‐OMAT V Ready‐Pack (Eastman Kodak Company, Rochester, NY) radiographic films of 35cm×43cm, for comparison. Since the absolute position of each leaf in these tests depends on the precision achieved during the MLC leaf calibration procedure, we took an additional step designed to account for the residual errors in the leaf calibration on the Bayouth test with radiographic films. Prior to executing the test patterns, a full MLC calibration was performed following the manufacturer's guidelines. Next, a film was irradiated using the Bayouth test with 50 MU per field, and its results in the area of the 27 inner leaves were taken as reference. The film was scanned using a VIDAR VXR‐16 Dosimetry Pro Advantage Scanner (VIDAR System Corporation, Herndon, VA) and analyzed using the RITv5.2 software (Radiological Imaging Technology, Colorado Springs, CO). In order to avoid errors caused by the field edge in the Bayouth test analysis, the first and last rows were discarded from the comparison and only the 25 innermost ones were analyzed. Based on the analysis of this reference pattern, a reference response matrix was obtained with the values of the initial leaves positions in order to eliminate the residual inaccuracy of the MLC calibration process from subsequent measurements.

The four verification test patterns with leaf position errors were then irradiated onto films, scanned, and analyzed using the same procedure. The leaf positions were obtained according to the standard Bayouth procedure and compared with the known predetermined position errors. In order to eliminate the effect of the residual leaf position errors during the MLC calibration, the reference response matrix was subtracted from the leaf position matrix obtained for each one of the four verification test patterns.

## III. RESULTS

**Figure 4:**
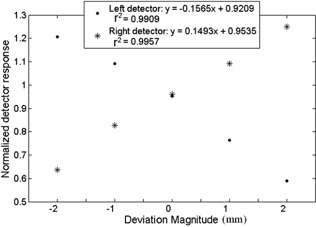
Partial volume response curves for the ionization chambers at the left and right sides of the central rectangle, under the central leaves.

All response curves to partial volume irradiation were similar to those shown in Fig. 4, corresponding to the ionization chambers in the 14th row, under the left and right side of the central rectangular field, and showed a linear behavior with small variations in the linear adjusted numerical parameters.

**Figure 5:**
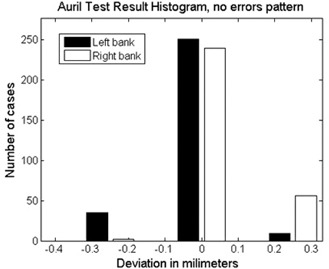
Result of AURIL software analysis of MLC for the 2D detector array exposed to a pattern without intentional errors.

**Figure 6:**
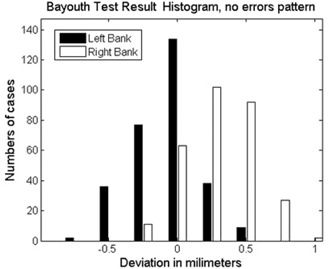
Illustration of the results using the Bayouth test analysis module within the RIT software for a film exposed to a pattern without intentional errors.

When the regular test patterns were irradiated over the PTW‐729 and over the films, it was found that the MLC positioning errors were within the tolerance established by the manufacturer. For AURIL, the positioning errors were in the range of ±0.4 mm (Fig. 5), while for the Bayouth method, these values were in the range of ±1 mm (Fig. 6).

**Figure 7:**
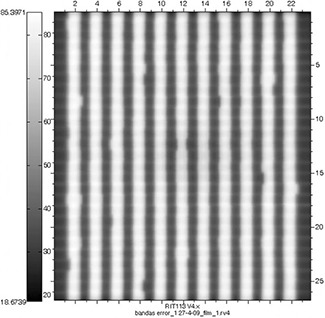
Film of one of the four validation patterns with intentional errors.

**Figure 8:**
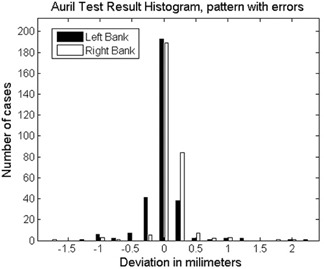
Errors detected by AURIL in the validation pattern corresponding to Fig. 7.

**Figure 9:**
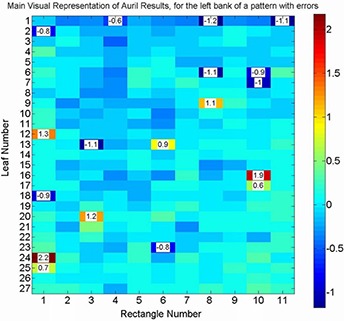
Graphic representation of the errors detected by AURIL software for the left leaves bank, in one of the validation pattern with errors corresponding to Fig. 7. The columns correlate with the rectangular beams in the pattern and contain 27 fields for the leaves' positions. Deviations equal to or larger than 0.6 mm are emphasized with their numerical values.

**Figure 10:**
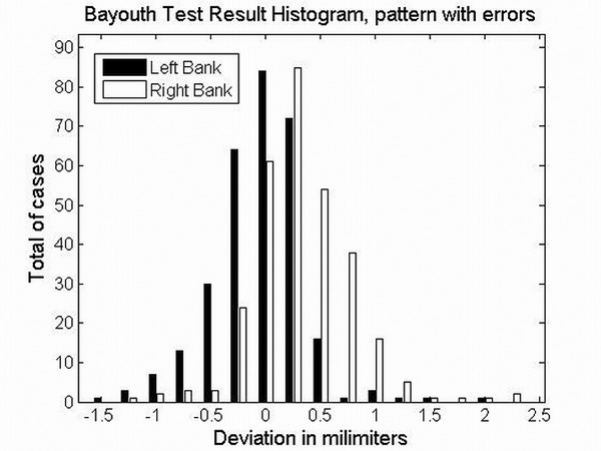
Errors in the validation pattern corresponding to Fig. 6, detected in the Bayouth test with films analyzed by the RIT software.

Figure 7 shows the film image of one of the verification test patterns with intentionally introduced MLC leaf positioning errors. Figure 8 shows the histograms of the errors detected by AURIL for this pattern when irradiated upon the PTW‐729. Figure 9 shows another graphic representation of the positioning errors from one of the MLC banks, as an additional tool within the AURIL program. Figure 10 shows the histogram of the MLC leaf positioning errors detected with the Bayouth test included in the RIT software for this same pattern.

Analyzing four different verification test patterns, it was found that AURIL detected the total number of intentionally introduced errors, as well as the direction of the deviations, as summarized in Table 1. AURIL binned values between 0.6 mm and 1.4 mm (0.97±0.17) to “1 mm” errors, values between 1.5 mm and 2.3 mm (1.84±0.16) to “2 mm” errors, and values of 2.7 mm and 2.8 mm to “3 mm” errors. The threshold for an error to be considered as “detected” was 0.6 mm. This criterion was adopted based on the minimum value obtained for “1 mm” deviations. Seven 0.6 mm deviation values and one 0.7 mm deviation value that did not correspond to predetermined errors were reported. Seven of these eight false errors were located on the field edge. These errors would have been among those discarded in the film‐based test.

Table 2 shows the errors detected by the Bayouth test performed with film for the same four verification test patterns. The total of the intentionally introduced errors larger than 1 mm were detected. The number of 1 mm detectable errors proved to be lower than that detected with the PTW‐729 and AURIL, and highly dependent on the established threshold value that defined a deviation as an “error”. The number of errors reported increases with a lower threshold value. There were a number of deviations reported in positions that neither corresponded to intentionally introduced errors, nor were they easily visible in the films. These deviations increased quickly with a lower threshold value, as shown in Table 2. The number of errors detected by the Bayouth method is also dependent on the definition by the operator of the central leafs axis position.

**Table 2:** Summary of errors and threshold dependency reported by the Bayouth test with film.

Detection threshold used (mm)	0.6	0.7	0.8	0.9	1.0
Total reported errors corresponding to those intentionally introduced	90	87	80	70	65
Reported errors not introduced and not visible in the film	324	239	147	66	31

Taking into account the reference matrix and its subtraction in the patterns with errors, the analysis showed higher specificity and similar sensitivity for low threshold values, causing a slight sensitivity decrease for high‐threshold values, as can be seen in Table 3. The methodology used by AURIL automatically takes into account the residual errors of the initial MLC calibration, since it is built into the initial calibration process.

**Table 3:** Summary of errors and threshold dependency reported by the Bayouth test with subtraction of the baseline.

Detection threshold used (mm)	0.6	0.7	0.8	0.9	1.0
Reported errors corresponding to those intentionally introduced	93	86	74	66	59
Reported errors not introduced and not visible in the film	58	14	5	0	0

## IV. DISCUSSION

The ideal MLC quality control method should be able to reveal slight differences between the prescribed positions and the actual positions of the leaves, over all the range of positions. Preferably, it should be simple, automatic, and independent of extraneous factors.

The Bayouth test with radiographic films allows a quantification of the leaf deviations, but since it uses the midpoint between a dosimetric peak and the adjacent valley to determine the field edge, it is possible to have opposite errors that do not alter the central position of a peak and the adjacent valleys. Under these circumstances, errors in leaf positioning would not be detected. Besides, the presence of a leaf position error introduces a shift in the dosimetric center of the adjacent peak and valley that may cause a false error report in the opposite side of that particular field or valley. In addition, our experience on the analysis of the Bayouth test using the RIT software indicates that its result is highly dependent on the choice of the central axis, and that an error threshold of less than 1 mm leads to an increasing number of false errors. Moreover, film dosimetry demands a high level of care, adding a burden that does not contribute to the value of the test. Also, the decreasing supply of film and photographic chemicals makes it necessary to find other alternatives.^(^ ^5^ ^,^ ^6^ ^)^

The subtraction of the reference matrix appears to increase the specificity for lower threshold values of errors in the Bayouth test. This is consistent with the hypothesis that the residual leaves calibration errors, while lower than 1 mm, remains quite stable on near consecutive measurements.

The detector response dependence as a function of its irradiated volume fraction by a radiation beam allows the implementation of our algorithm to any other type of detector arrays, as long as they display a partial volume response. Although in our work only the 27 inner leaves were targeted, it is clear that the totality of the MLC can thus be evaluated with straightforward shifts of the PTW‐729 in the gun‐target direction.

The MLC_Fastchecker software uses a calibration somehow similar to ours, based on variations in the position of adjacent fields in order to identify deviations at the field edge. These deviations are seen in the response of the detectors that agree with the field boundaries.^(^ ^10^ ^)^ Thus, deviations in leaf positioning are quantified. Since two adjacent fields cover a detector, the method is not able to indicate which bank of leaves is responsible for a particular error. A one‐leaf error could be compensated by an error of the opposite leaf and neither of them would be detected. All 729 detectors are characterized by a unique calibration parameter.

The analysis developed with AURIL software to be used with the PTW‐729 responds directly to position variation of the field boundaries being irradiated. Unlike the MLC_Fastchecker, each leaf bank can be evaluated independently, without opposing errors being cancelled. In addition, for each detector, the reading corresponding to the initial independent calibration is registered on a matrix for future use. The few false errors reported were found mostly at the field edges, where scattering conditions are more extreme.

The initial system calibration time takes approximately 60 minutes, which includes the device setup and the irradiation of nine patterns with 11 fields. This initial calibration is valid for as long as the MLC calibration does not change. The acquisition time for a control test is about 20 minutes. Considering the preliminary results of ongoing studies, the number of MU for each field could be significantly reduced, shortening these times without affecting the quality of the results.

## V. CONCLUSIONS

This work proposes a new method for quality control of a MLC using a PTW‐729 ion chamber array. The principle of varying signal response of a volumetric detector to partial irradiation enables this method to correlate the signal with the variations of the radiation beam edges and, therefore, to detect deviations in the expected position of the leaves with increased spatial resolution.

The method is easy to implement and does not exhibit many of the problems related to the use of radiographic films. It was possible to detect the total number of predetermined deviations and to quantify them at their correct frequency, with a minimum number of false errors. It is easy to modify to cover the whole MLC by doing the test in two stages. AURIL software may become a useful tool for the routine quality control of MLCs at radiotherapy departments.

## ACKNOWLEDGMENTS

The authors wish to thank Silvia Zunino, PhD, for her priceless help and encouragement to conduct this research.

R. DÍaz Moreno wishes to thank Rodolfo Alfonso, PhD, and Carlos Sánchez Catasús, PhD, for valuable teaching and inspiration. He gratefully acknowledges the support of the Instituto de Neurologia y Neurociencia, Cuba, and of Instituto de Radioterapia ‐ Fundación Marie Curie, Argentina.

Part of this work was carried our under the framework of a co‐ordinated research project E.2.40.15 with the International Atomic Energy Agency (IAEA, Vienna). All authors would like to thank the IAEA for the support it provided under this project.

## References

[1] Boyer A , Biggs P , Galvin J , et al. AAPM Report No. 72: Basic applications of multileaf collimators, report of Task Group No. 50 Radiation Therapy Committee. Madison, WI: AAPM; 2001.

[2] Yan G , Liu C , Simon T , Peng LC , Fox C , Li J . On the sensitivity of patient‐specific IMRT QA to MLC positioning errors. J Appl Clin Med Phys. 2009;10(1):120–28. 10.1120/jacmp.v10i1.2915 PMC5720508 19223841

[3] Rangel A and Dunscombe P . Tolerances on MLC leaf position accuracy for IMRT delivery with a dynamic MLC. Med Phys. 2009;36(7):3304–09. 19673226 10.1118/1.3134244

[4] Bayouth JE , Wendt D , Morrill SM . MLC quality assurance techniques for IMRT applications. Med Phys. 2003;30(5):743–50. 12772980 10.1118/1.1564091

[5] Poppe B , Blechschmidt A , Djouguela A , et al. Two‐dimensional ionization chamber arrays for IMRT plan verification. Med Phys. 2006;33(4):1005–15. 16696477 10.1118/1.2179167

[6] Van Esch A , Clermont C , Devillers M , Iori M , Huyskens DP . On‐line quality assurance of rotational radiotherapy treatment delivery by means of a 2D ion chamber array and the Octavius phantom. Med Phys. 2007;34(10):3825–37. 17985628 10.1118/1.2777006

[7] Wiezorek T , Banz N , Schwedas M , et al. Dosimetric quality assurance for intensity‐modulated radiotherapy. Feasibility study for a filmless approach. Strahlenther Onkol. 2005;181(7):468–74. 15995841 10.1007/s00066-005-1381-z

[8] Yang Y and Xing L . Using the volumetric effect of a finite‐sized detector for routine quality assurance of multileaf collimator leaf positioning. Med Phys. 2003;30(3):433–41. 12674244 10.1118/1.1543150

[9] Spezi E , Angelini AL , Romani F , Ferri A . Characterization of a 2D ion chamber array for the verification of radiotherapy treatments. Phys Med Biol. 2005;50(14):3361–73. 16177515 10.1088/0031-9155/50/14/012

[10] Salz H . Checking MLC accuracy with the Array Seven29 (PTW Frieburg) and the MLC Fastchecker program. Jena University Hospital, Department of Radiotherapy and Radiooncology, Bachstrasse, Jena, Germany Available from: http://www.ptw.de/mlc_fastchecker.html.

[11] Bayouth JE . Siemens multileaf collimator characterization and quality assurance approaches for intensity‐modulated radiotherapy. Int J Radiat Oncol Biol Phys. 2008;71(1‐Suppl):S93–S97. 18406947 10.1016/j.ijrobp.2007.07.2394

